# The Accuracy of Height Prediction Equations in Greek Patients: A Cross-Sectional Study

**DOI:** 10.3390/nu16132062

**Published:** 2024-06-28

**Authors:** Anna Papadopoulou, Dimitrios Karayiannis, Georgatza Dimitra, Zoe Pafili

**Affiliations:** Department of Clinical Nutrition, Evangelismos General Hospital, 10676 Athens, Greece; annapapad6983@gmail.com (A.P.); dkarag@hua.gr (D.K.); dimitragrg@hotmail.com (G.D.)

**Keywords:** height, stature, Greek patients, Malnutrition Universal Screening Tool (MUST), equations

## Abstract

In clinical settings, standing height measurement is often difficult to perform due to patients’ inability to stand upright. Height prediction equations derived from measurements of the length of other body segments have been published; however, they are not readily applicable to all populations since ethnic differences affect the relationship between standing height and body segment length. This cross-sectional study aimed to examine the accuracy of height prediction using the Malnutrition Universal Screening Tool (MUST) height predictive equations among Greek patients and to develop new, nationally representative equations. The study population consisted of 1198 Greek adult outpatients able to stand upright without assistance and without medical conditions that affected their height. Standing height, ulna length, knee height and demi-span measurements were obtained from 599 males and 599 females. Patients were stratified into age groups of <55 and ≥55 years, <60 and ≥60 years and <65 and ≥65 years according to the categories indicated by the MUST for height prediction from alternative measurements. There were positive correlations between standing height and ulna length and knee height and demi-span length (*p* < 0.001) in both sexes and all age categories. A strong correlation was observed between the measured and predicted standing height using ulna length (rho = 0.870, *p* < 0.001), knee height (rho = 0.923, *p* < 0.001) and demi-span length (rho = 0.906, *p* < 0.001). The average difference between the MUST indicative equations’ height predictions from alternative measurements and actual height was −3.04 (−3.32, −2.76), −1.21 (−1.43, −0.988) and 2.16 (1.92, 2.41), respectively. New height prediction equations for Greek patients were identified, with the predicted values closer to the measured standing heights than those predicted with the MUST indicative equations for height prediction from alternative measurements.

## 1. Introduction

Collecting anthropometric data from patients in the clinical setting is crucial to accurately assessing patients’ clinical and nutritional status, ensuring they receive adequate care and diet therapy. More specifically, precise height measurement holds significant relevance as it enables calculation of the Βody Μass Ιndex (BMI). BMI is a component of almost all nutritional screening tools and is a crucial parameter for malnutrition diagnosis according to the European Society of Clinical Nutrition and Metabolism (ESPEN) [1,2]. Furthermore, the equations used to predict Basal Metabolic Rate (BMR) and estimate nutritional requirements also encompass height as a parameter [3], whereas precise height measurement holds considerable significance among hospitalized patients, often extending beyond nutritional assessment since it is necessary for the estimation of body surface area to determine optimal drug dosages [4], the estimation of renal function for patients undergoing hemodialysis [5] and tidal volume in critically ill patients [6,7].

However, for hospitalized patients, obtaining precise height measurement using a stadiometer is often not feasible, and research demonstrates the overestimation of height when self-reported [8]. Hence, there arises a necessity for practical alternatives to accurately assess patients’ height [9]. This challenge is addressed using height calculation equations that utilize measurements of more accessible body segments that correlate with height, such as ulna length [10,11,12], knee height [13,14,15,16,17,18,19], tibia length [20], demi-span [21,22], arm length [23] and arm span [24,25]. One of the oldest and most frequently utilized sets of equations is presented by the Malnutrition Universal Screening Tool (MUST) [26]. The MUST indicative equations for height prediction from alternative measurements, which were validated in hospitalized patients in England, offer flexibility in height prediction in using three distinct possible measurements: ulna length, knee height and demi-span length. Furthermore, equations have been developed from the NHANES study based on validation within the US population [27].

It is acknowledged that anthropometric measurements, such as height, and the relationship between standing height and body segment length exhibit variation across populations [24,28,29], leading to differences in the equation-predicted height among various ethnic groups [16]. Height also tends to decrease with advancing age, and research suggests that this decline is not consistent over time [30]. Since the MUST is one of the most frequently used tools for nutritional risk screening, several studies have attempted to assess the precision and suitability of the MUST indicative equations for height prediction from alternative measurements for several ethnicities [11,31,32,33], while some others have questioned their applicability to various ethnic groups [32,33]. Additionally, the application of the equations to different age groups has been poorly investigated [11]. Since specific equations for the Greek population do not exist, the need arises not only to evaluate the suitability of the MUST indicative equations for height prediction from alternative measurements for the Greek population but also to assess their accuracy with respect to the age of the patients. This study aims to evaluate the accuracy of the published MUST indicative equations for height prediction from alternative measurements in predicting height based on ulna length, knee height and demi-span length in Greek adult outpatients. Additionally, we endeavor to develop height estimation equations using measurements of alternative body segments specific to the Greek population.

## 2. Materials and Methods

The current cross-sectional study, conducted at the University General Hospital of Larissa between September 2017 and December 2021, included both inpatients from hospital units and outpatients visiting the clinics. The study focused on adult participants of both genders who were capable of standing upright. The exclusion criteria were male individuals under 21 years old due to the potential incompleteness of their development, patients with musculoskeletal or orthopedic conditions that hindered their ability to maintain an upright posture and those with metabolic disorders impacting their full height growth. Foreign nationals and patients in isolation were also excluded. Participation in the study required the patients to provide informed consent after receiving detailed information about the study’s objectives from the research team. The study received ethical approval from the University of Thessaly Medical. School Ethics Committee (11/22-07-2016).

The participants’ standing posture was assessed using a stable stadiometer, Charder HM200P (Charder Electronic Co., Ltd., Taichung City, Taiwan) to the nearest 0.1 cm. Three long bone length measurements were also made, ulna length, half of the arm span and knee height, using a Seca tape measure 203 (Seca gmbh & Co., Hamburg, Germany). All the measurements were performed by a trained nutritionist during the early morning hours to ensure the most accurate assessment of the participants’ standing position. Each measurement was conducted twice, and if the disparity between the initial two measurements exceeded 1 cm (cm), a third measurement was taken. The final value for each parameter was derived from the average of the two or three measurements. The methodology adhered to the standards set by the British Association for Parenteral and Enteral Nutrition (BAPEN) to ensure consistency and reliability in the measurement process [26].

For standing height measurement, patients removed any hats and footwear and unstyled their hair, and they were asked to stand upright with relaxed hands, pressing their whole foot against the base and resting their heels on the back. Attention was paid to aligning the patient’s head using the Frankfort method, ensuring their eyes were level with their upper earlobes without leaning back. Height was obtained by gently lowering the sliding plate until it touched the top of the patient’s head.

To measure ulna length, patients removed any jewelry or watches that might affect accuracy and were then asked to bend their left arm, placing their palm on their chest with their fingers pointing towards the opposite shoulder. The distance between the bony prominence at the elbow tip (ulna) and the midpoint of the protruding wrist bone was measured to the nearest 0.1 cm. For knee height measurement, patients bent their left leg at a right angle while sitting on a chair without shoes. The investigator held the measuring tape between their middle and ring fingers, positioning the zero mark under the fingers. Placing their hand parallel to the patient’s thigh, approximately 4 cm behind the front of the knee, the tape was extended straight down alongside the foot, aligning with the ankle bone and the base of the heel. To measure half of the arm span, the midpoint of the cervical notch was located and marked. Patients removed any jewelry or watches and raised their right hand horizontally to their shoulder, with their wrist straight, palm facing forward and fingers spread apart. The distance from the midpoint of the cervical notch to the middle finger of the patient’s right hand was measured.

All statistical analyses were performed using the Jamovi program(www.jamovi.org). Data were checked to ensure that there were no outliers in the demi-span length, knee height, ulna length or height measurements. Continuous variables with a normal distribution were described using means and standard deviation, while continuous variables without a normal distribution were described as medians and interquartile intervals. Qualitative variables were described with absolute numbers and frequencies. The normality of the distribution was evaluated using the Shapiro–Wilk test. Based on the variable distribution, correlations between standing height and ulna length, knee height and demi-span length were evaluated using either Pearson’s or Spearman’s correlation tests. Additionally, the correlation between measured height and height predicted from ulna length, knee height, and demi-span length based on the MUST indicative equations for height prediction from alternative measurements was also assessed using the same methods. Linear regression was used to identify new height predictive equations based on ulna length, knee height and demi-span length in age groups corresponding to the MUST indicative equations for height prediction from alternative measurements for each variable. Scatter plots were used to graphically represent the correlation between the measured and estimated heights based on the MUST indicative equations for height prediction from alternative measurements and the correlation between measured height and measured ulna length, knee height and demi-span length, respectively. The agreement between measured and predicted height (from ulna length, knee height and demi-span length measurements), respectively, was evaluated for both sexes and different age categories through Bland–Altman plots. Differences in the predicted means between the two methods were calculated using a paired t-test. The level of significance was set at 0.05.

## 3. Results

### 3.1. Demographics

In total, 1998 patients were evaluated, of whom 599 (50%) were male. Male patients were aged 64 (±22.5) years old and their height was 170 (±7.59) cm, while females were aged 65 (±22) years old and their height was 157 (±9.5) cm. Details of the patients’ characteristics are presented in Table 1 according to sex and the age categories used by the MUST indicative equations for height prediction from alternative measurements for predicting height from demi-span length (<55 and ≥55 years), knee height (<60 and ≥60 years) and ulna length (<65 and ≥65 years) for both sexes, respectively.

### 3.2. Correlation Analysis

**Figure 1:**
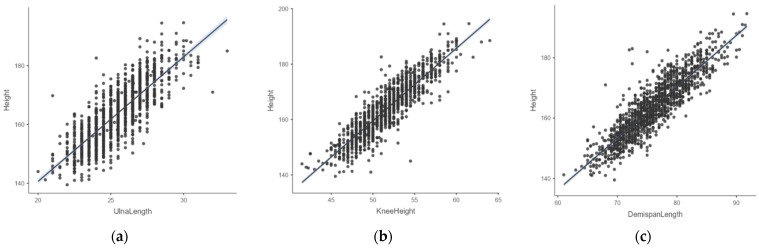
Scatter plots showing the correlation between measured height and ULN, KNH and DSL, respectively. (**a**) Correlation between height measurement and ulna length measurement, (**b**) correlation between height measurement and knee height measurement, (**c**) correlation between height measurement and demi-span length measurement. All height and length measurements reported in cm. Abbreviations: ULN, ulna length in cm; KNH, knee height; DSL, demi-span length.

Table 2 displays the positive correlation (*p* < 0.001) between measurements of height and ulna length, knee height and demi-span length, respectively, for both sexes and all age categories. The correlation coefficients between height and ulna length were always lower than those for knee height or demi-span length, while they were even lower in females compared to males. The correlation between height and various body measurements is illustrated in Figure 1. The strongest correlation was observed between height and knee height.

### 3.3. Mean Height Measurements

The mean height measurement for the study population was 164 (±9.93) cm. The mean estimated height based on ulna length, knee height and demi-span measurements using the MUST indicative equations for height prediction from alternative measurements was 167 (±8.38) cm, 165 (±8.71) cm and 162 (±8.53) cm, respectively.

### 3.4. Estimated Height Using the MUST Indicative Equations for Height Prediction from Alternative Measurements

**Figure 2:**
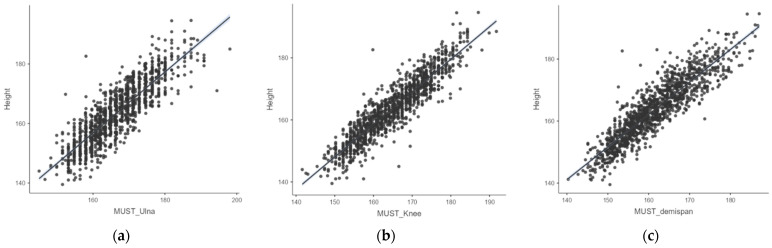
Scatter plots showing the correlation between measured height and estimated height from ULN, KNH and DSL, respectively. (**a**) Correlation between measured height and estimated height from ulna length measurement using the MUST indicative equations for height prediction from alternative measurements, (**b**) correlation between measured height and estimated height from knee height measurement using the MUST indicative equations for height prediction from alternative measurements, (**c**) correlation between measured height and estimated height from demi-span length measurement using the MUST indicative equations for height prediction from alternative measurements. All height and length measurements reported in cm. Abbreviations: ULN, ulna length in cm; KNH, knee height; DSL, demi-span length.

Equations for the prediction of height based on ulna length, knee height and demi-span length were identified using linear regression for each sex using age categories corresponding to the MUST indicative equations for height prediction from alternative measurements, as displayed in Table 3. The predictive value was better for equations using knee height compared to those using demi-span and ulna length. The corresponding MUST indicative equations for height prediction from alternative measurements by sex and age category for each independent variable are also displayed in Table 3. Strong correlations were found between the measured and estimated heights using ulna length, knee height and demi-span length (all *p* < 0.001), as displayed in Figure 2.

### 3.5. Agreement Analysis—Bland–Altman Plots

**Figure 3:**
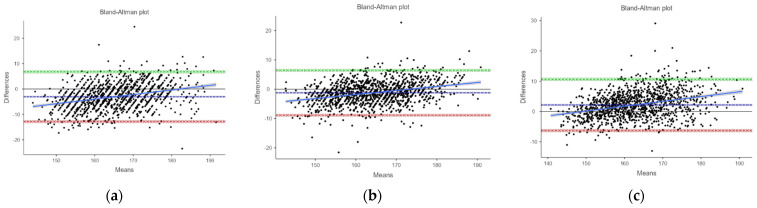
Bland-Altman plot showing the agreement between measured height and estimated height from ULN, KNH and DSL, respectively. (**a**) Bland–Altman plot showing the agreement between measured height and estimated height from ulna length measurement using the MUST indicative equations for height prediction from alternative measurements, (**b**) Bland–Altman plot showing the agreement between measured height and estimated height from knee height measurement using the MUST indicative equations for height prediction from alternative measurements, (**c**) Bland–Altman plot showing the agreement between measured height and estimated height from demi-span length measurement using the MUST indicative equations for height prediction from alternative measurements.

**Figure 4:**
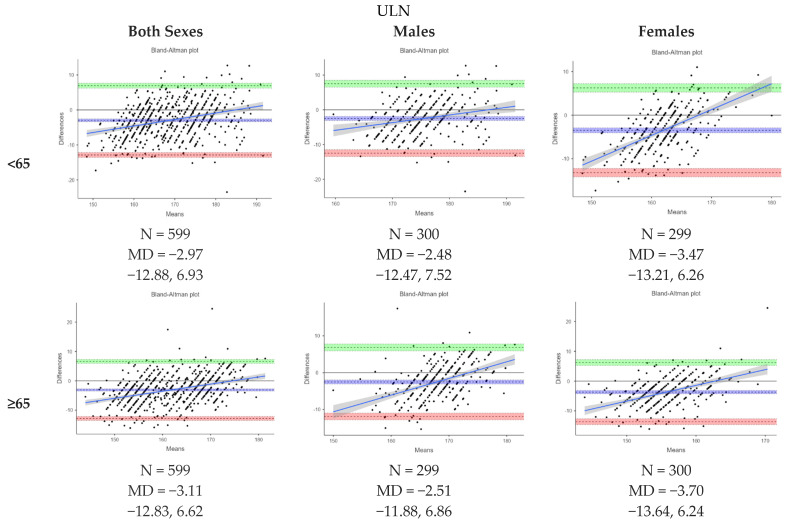
Bland-Altman plot showing the agreements between measured height and estimated height from ulna length by sex and age category based on MUST indicative equations for height prediction from alternative measurements. All height measurements reported in cm and age reported in years. Abbreviations: ULN, ulna length in cm. All height measurements reported in cm. Abbreviations: ULN, ulna length in cm; KNH, knee height; DSL, demi-span length.

**Figure 5:**
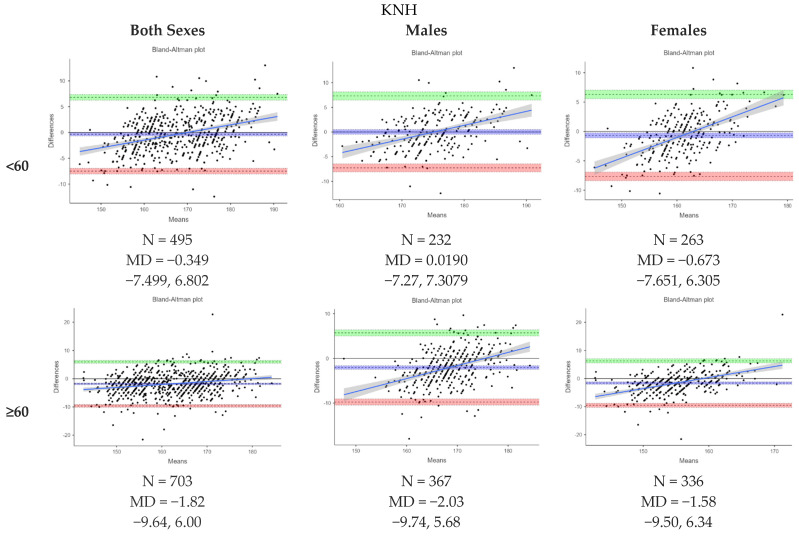
Bland-Altman plot showing the agreements between measured height and estimated height from knee height by sex and age category based on MUST indicative equations for height prediction from alternative measurements. All height measurements reported in cm and age reported in years. Abbreviations: KNH, knee height.

**Figure 6:**
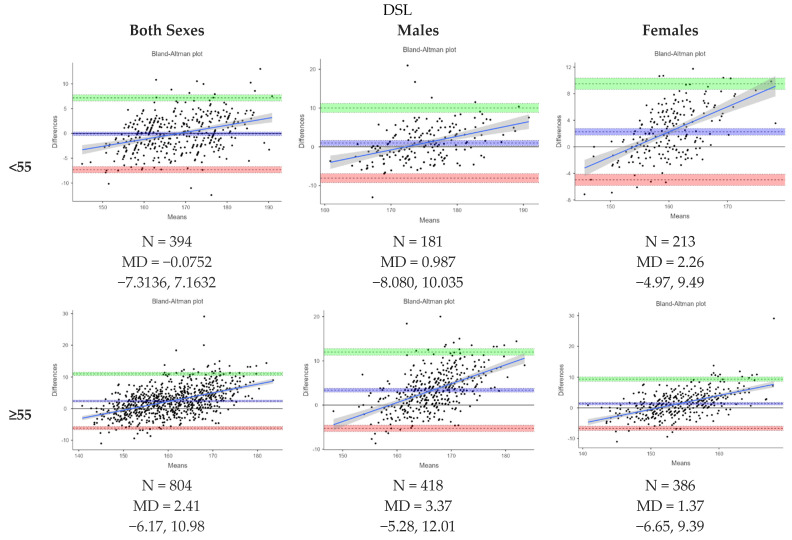
Bland-Altman plot showing the agreements between measured height and estimated height from demi-span length by sex and age category based on MUST indicative equations for height prediction from alternative measurements. All height measurements reported in cm and age reported in years. Abbreviations: DSL, demi-span length.

Figure 3 presents Bland–Altman plots depicting the agreement between the measured and estimated heights using ulna length, knee height and demi-span length according to the MUST indicative equations for height prediction from alternative measurements. The mean difference (MD) between the measured and estimated heights from ulna length was −3.04 (−3.32, −2.76) cm, from knee height, it was −1.21 (−1.43, −0.988) cm, and from demi-span length, it was 2.16 (1.92, 2.41) cm. The levels of agreement varied between methods. Figure 4, Figure 5 and Figure 6 further analyze the agreement by sex and age category for the estimated heights from ulna length and knee height, respectively, using the MUST indicative equations for height prediction from alternative measurements. The mean differences between the measured and estimated heights vary across age categories and sexes, showing different levels of agreement.

These results underscore the utility of different body measurements for estimating height and emphasize the importance of considering demographic factors in height estimation models.

## 4. Discussion

In this study, a comprehensive dataset of measurements from several body segments (long bone lengths) of Greek patients was gathered in order to evaluate the applicability of the MUST indicative equations for height prediction from alternative measurements in predicting stature across genders and different age groups within the Greek population [26]. In addition, the present study evaluated the capability to accurately predict stature from measurements of different body segments with the aim of refining equations with enhanced predictive value for the Greek population. The results demonstrate favorable alignment between the actual height measurements and the predicted heights derived from the newly identified equations, with the optimal predictive capability observed for the knee height measurements.

Several alternative methods have been proposed in the available literature for predicting height, utilizing measurements of different body segments across ethnicities and age categories. The results of the present study are consistent with existing literature regarding the correlation between height and measurements of other body segments such as ulna length, knee height and demi-span length [10,11,12,13,14,15,16,18]. Our findings suggest a stronger correlation between height and knee height or demi-span length compared to ulna length, although ulna length is extensively studied due to its ease of measurement [10,11,12]. More specifically, the smaller the mean difference between the predicted and measured height, the better the predictability of the equations for the population in question, and thus this study’s findings suggest the superiority of knee height measurement in predicting height.

Regarding the superiority of knee height compared to other body segments, such as ulna length, arm span, demi-span, tibia length and fibula length, in predicting height, the findings of this study are in agreement with the results of a cross-sectional study examining the accuracy of various different equations in predicting height for African hospitalized adult patients [32]. In this study by Williamson et al., it was demonstrated that only a single equation published by Chumlea et al. which was based on knee height measurements showed a good predictive capability for the measured heights within the tested sample [17]. Likewise, a study investigating the efficacy of arm circumference and the four-point method in predicting height for critically ill patients demonstrated that the most effective method was the four-point approach, incorporating measurement of knee height [34]. Previous research conducted by Pini and colleagues has already demonstrated the superiority of knee height measurement as the optimal method for estimating height when accurate measurement cannot be carried out [35]. However, Auyeung et al. gathered a comprehensive sample of elderly Chinese patients over 65 years old and showed a favorable agreement between height prediction from knee height and ulna length or fibula measurement, suggesting the alternative use of these measurements if knee height is not accessible [10]. Finally, in addition to the MUST indicative equations for height prediction from alternative measurements, several equations incorporating knee height measurement have been published due to its outstanding predictive accuracy [15,19].

The MUST indicative equations for height prediction from alternative measurements were derived from measurements of patients in England and have afterwards been tested for their accuracy in populations from different ethnic backgrounds [11,31,32,33]. As demonstrated in this study, the MUST indicative equations for height prediction from alternative measurements overestimate the stature of Greek patients, although a satisfactory level of alignment between the predicted and measured heights was demonstrated. Barbosa et al. evaluated the accuracy of the MUST tool indicative equations for height prediction from alternative measurements by using ulna length measurements and showed favorable concordance between the predicted and measured heights for both English and Portuguese patients [11]. The reported alignment exceeds the concordance observed between the measured and predicted heights derived from ulna length found in the present study. In contrast, a cross-sectional study examining the agreement between measured and predicted heights using ulna length according to the MUST indicative equations for height prediction from alternative measurements for Asian and Black adult populations demonstrated significant differences between measured and predicted height in these populations [31,33]. Similar findings emerged from a study examining adult African patients, showing the poor efficacy of the MUST indicative equations for height prediction from alternative measurements in accurately predicting height [32].

The decline of stature with advancing age is of no doubt, and research suggests this decline is not consistent [30]. Therefore, in terms of the accuracy of the MUST indicative equations for height prediction from alternative measurements or other equations aiming to predict height from measurements of various body segments, age should be taken into consideration as a confounding factor [11]. This issue has been addressed by some studies that have aimed to investigate the applicability of height prediction equations across different age cohorts, typically older adults or elderly populations [10,27], while other studies have focused on evaluating the accuracy of height predicting equations across different ethnicities and between genders while failing to examine differences between different age groups [33].

Compared to the MUST indicative equations for height prediction from alternative measurements, the application of the equations derived in the present study resulted in better agreement with the measured heights when knee height and demi-span length were used as factors for age categories <60 and <55 years old in both sexes, which was not the case for equations based on ulna length. These findings come in agreement with Barbosa et al., who also demonstrated the poor predictive capability of the MUST indicative equations for height prediction from alternative measurements based on ulna length in younger individuals [11].

This study was the first systematic attempt to assess whether the MUST indicative equations for height prediction from alternative measurements based on ulna length, knee height and demi-span length can be applied to the Greek population with accuracy. For that reason, we included a large sample of patients from a single hospital serving one of the largest prefectures in the country, with the aim of including a fairly representative sample of the population. In addition, the sample collection and measurements were conducted by a single trained dietician with the aim of minimizing inter-observer errors. Furthermore, all necessary measures were undertaken to ensure consistency and reliability in the measurement process for height and all body segments.

Based on the collected data, new equations based on knee height and demi-span length were devised with better predictive capacity for Greek patients than the respective MUST indicative equations for height prediction from alternative measurements. This is the first attempt to identify specific height prediction equations for the Greek population.

However, this study bears several limitations that must be acknowledged. The patient sample was exclusively collected from a single hospital in Greece, which affects the generalization of the findings. Despite the exclusive inclusion of participants of Greek origin, the sample cannot be considered representative of the Greek population. Nevertheless, it should be noted that all participants were either outpatients or inpatients, constituting a representative sample of hospitalized patients to whom the study’s results aim to be applied. Furthermore, it is well known that stature is not only influenced by sex and age but also by several environmental factors, such as nutrition, disease and socio-economic status [36]. These factors were not considered in the development of the height prediction equations for Greek patients. Finally, an important limitation of the present study is the omission of weight measurement and therefore the inability to calculate the patients’ BMI. However, the research demonstrates that deviation between measured and estimated heights based on equations is not related to BMI [11].

There is no doubt that the correlation between stature and the length of body segments such as ulna length, knee height and demi-span varies among different ethnicities, genders and age groups. Therefore, height prediction equations should take these parameters into consideration. Further multicenter studies should confirm the accuracy of the derived equations in predicting height in the Greek population.

## 5. Conclusions

In conclusion, the correlation between stature and measurements of various body segments can be particularly helpful in hospital settings where accurate measurement of height is often impossible. The strong correlation observed between knee height and stature suggests that the utilization of knee height is the most reliable variable for predicting height in younger Greek patients.

## Figures and Tables

**Table 1:** Characteristics of subjects according to sex and age category.

**Males**							
	**Ν**	**<55**	**≥55**	**<60**	**≥60**	**<65**	**≥65**
	599 (50%)	181 (15.1%)	418 (34.9%)	232 (19.4%)	367 (30.6%)	300 (25%)	299 (25%)
Age (years)	64 (22.5)	43 (17)	71 (13)	47.5 (17)	72 (11)	51 (18)	74 (8)
Height (cm)	170 (±7.59)	176 (±6.77)	168 (±6.84)	175 (±6.59)	167 (±6.57)	174 (±6.73)	167 (±6.39)
Ulna length (cm)	26.5 (2)	27.5 (2)	26.5 (1.5)	27 (2)	26.5 (1.5)	27 (2)	26.5 (1.5)
Knee height (cm)	53.5 (3.5)	55.2 (±2.76)	53 (3.5)	55.1 (±2.72)	53 (3)	54.6 (4)	52.5 (3.5)
Demi-span length (cm)	79.4 (±4.26)	82.2 (±3.84)	78.2 (±3.88)	81.9 (±3.92)	77.9 (±3.72)	81.3 (±3.94)	77.6 (±3.73)
**Females**							
	**Ν**	**<55**	**≥55**	**<60**	**≥60**	**<65**	**≥65**
	599 (50%)	213 (17.8%)	386 (32.2%)	263 (22%)	336 (28%)	299 (24.9%)	300 (25%)
Age (years)	65 (22)	43 (14)	69 (9)	47 (15)	70 (9)	49 (16.5)	71 (7)
Height (cm)	157 (9.5)	161 (±6.44)	154 (8.57)	161 (±6.49)	154 (8.33)	160 (±6.49)	153 (8.2)
Ulna length (cm)	24 (1.5)	24.5 (1.5)	24 (1.69)	24.5 (2)	24 (1.5)	24.5 (2)	24 (1.5)
Knee height (cm)	49.3 (3)	50.1 (±2.51)	49 (3)	50.1 (±2.5)	49 (2.56)	50 (±2.49)	48.5 (2.5)
Demi-span length (cm)	72.8 (±3.7)	74 (5)	71.9 (±3.49)	74.3 (±3.53)	71.6 (±3.39)	74.2 (±3.56)	71.4 (±3.29)

Values are presented as means (±SD) or medians (IQRs) for parametric and non-parametric continuous variables, respectively. Values are presented as absolute numbers (%) for qualitative variables.

**Table 2:** Pearson’s or Spearman’s correlation coefficients between height measurement and ULN, KNH, DSL by sex and age category.

**Males**							
	**Ν**	**<55**	**≥55**	**<60**	**≥60**	**<65**	**≥65**
Ulna length	0.671 *	0.701 *	0.620 *	0.693 *	0.621 *	0.685 *	0.650 *
Knee height	0.825 *	0.819 *	0.802 *	0.828 *	0.790 *	0.819 *	0.787 *
Demi-span length	0.803 *	0.731 *	0.769 *	0.727 *	0.760 *	0.746 *	0.755 *
**Females**							
	**Ν**	**<55**	**≥55**	**<60**	**≥60**	**<65**	**≥65**
Ulna length	0.616 *	0.566 *	0.587 *	0.595 *	0.579 *	0.580 *	0.556 *
Knee height	0.786 *	0.849 *	0.778 *	0.845 *	0.763 *	0.835 *	0.758 *
Demi-span length	0.808 *	0.779 *	0.765 *	0.825 *	0.767 *	0.818 *	0.750 *

Pearson’s r is presented for correlations where both variables have normal distribution. Spearman’s rho (non-parametric equivalent) is presented for correlations where at least one variable did not have normal distribution* *p* < 0.001. Abbreviations: ULN, ulna length in cm; KNH, knee height; DSL, demi-span length.

**Table 3:** Linear regression equations to estimate height using ULN, KNH and DSL compared to the MUST equations for each variable, sex and age group.

			**Linear Regression**	**R**	**MUST Indicative Equations for Height Prediction from Alternative Measurements**	**R**
ULN	Males	<65	y = 95.57 + 2.91x	0.671	y = 79.29 + 3.60x	1
		≥65	y = 85.80 + 3.07x	0.663	y = 86.22 + 3.15x	1
	Females	<65	y = 87.48 + 2.96x	0.645	y = 95.60 + 2.77x	1
		≥65	y = 88.17 + 2.75x	0.554	y = 79.81 + 3.26x	1
			**Linear Regression**	**R**	**MUST Indicative Equations for Height Prediction from Alternative Measurements**	**R**
KNH	Males	<60	y = 64.62 + 2.01x	0.828	y = 72.10 + 1.87x	1
		≥60	y = 52.50 + 2.17x	0.802	y = 59.25 + 2.08x	1
	Females	<60	y = 50.97 + 2.19x	0.845	y = 67.73 + 1.87x	1
		≥60	y = 54.60 + 2.04x	0.749	y = 61.92 + 1.92x	1
			**Linear Regression**	**R**	**MUST Indicative Equations for Height Prediction from Alternative Measurements**	**R**
DSL	Males	<55	y = 69.51 + 1.29x	0.731	y = 67.77 + 1.30x	1
		≥55	y = 62.01 + 1.36x	0.770	y = 70.99 + 1.20x	1
	Females	<55	y = 47.32 + 1.53x	0.829	y = 62.05 + 1.30x	1
		≥55	y = 55.61 + 1.38x	0.765	y = 67.12 + 1.20x	1

All height and length measurements reported in cm and age reported in years. Abbreviations: ULN, ulna length in cm; KNH, knee height; DSL, demi-span length.

## Data Availability

The data that support the findings of this study are available on reasonable request from the corresponding author [Z.P].

## References

[1] Cederholm T., Jensen G.L., Correia M., Gonzalez M.C., Fukushima R., Higashiguchi T., Baptista G., Barazzoni R., Blaauw R., Coats A. (2019). GLIM criteria for the diagnosis of malnutrition—A consensus report from the global clinical nutrition community. Clin. Nutr..

[2] Kyle U.G., Kossovsky M.P., Karsegard V.L., Pichard C. (2006). Comparison of tools for nutritional assessment and screening at hospital admission: A population study. Clin. Nutr..

[3] Henry C.J. (2005). Basal metabolic rate studies in humans: Measurement and development of new equations. Public Health Nutr..

[4] Felici A., Verweij J., Sparreboom A. (2002). Dosing strategies for anticancer drugs: The good, the bad and body-surface area. Eur. J. Cancer.

[5] Schwartz G.J., Haycock G.B., Edelmann C.M., Spitzer A. (1976). A simple estimate of glomerular filtration rate in children derived from body length and plasma creatinine. Pediatrics.

[6] Crapo R.O., Morris A.H., Clayton P.D., Nixon C.R. (1982). Lung volumes in healthy nonsmoking adults. Bull. Eur. Physiopathol. Respir..

[7] Crapo R.O., Crapo J.D., Morris A.H. (1982). Lung tissue and capillary blood volumes by rebreathing and morphometric techniques. Respir. Physiol..

[8] Connor Gorber S., Tremblay M., Moher D., Gorber B. (2007). A comparison of direct vs. self-report measures for assessing height, weight and body mass index: A systematic review. Obes. Rev..

[9] Hickson M., Frost G. (2003). A comparison of three methods for estimating height in the acutely ill elderly population. J. Hum. Nutr. Diet..

[10] Auyeung T.W., Lee J.S., Kwok T., Leung J., Leung P.C., Woo J. (2009). Estimation of stature by measuring fibula and ulna bone length in 2443 older adults. J. Nutr. Health Aging.

[11] Barbosa V.M., Stratton R.J., Lafuente E., Elia M. (2012). Ulna length to predict height in English and Portuguese patient populations. Eur. J. Clin. Nutr..

[12] Ilayperuma I., Nanayakkara G., Palahepitiya N. (2010). A Model for the Estimation of Personal Stature from the Length of Forearm. Int. J. Morphol..

[13] Han T.S., Lean M.E. (1996). Lower leg length as an index of stature in adults. Int. J. Obes. Relat. Metab. Disord..

[14] Ritz P. (2004). Validity of measuring knee-height as an estimate of height in diseased French elderly persons. J. Nutr. Health Aging.

[15] Chumlea W.C., Guo S. (1992). Equations for predicting stature in white and black elderly individuals. J. Gerontol..

[16] Palloni A., Guend A. (2005). Stature prediction equations for elderly Hispanics in Latin American countries by sex and ethnic background. J. Gerontol. A Biol. Sci. Med. Sci..

[17] Chumlea W.C., Guo S.S., Steinbaugh M.L. (1994). Prediction of stature from knee height for black and white adults and children with application to mobility-impaired or handicapped persons. J. Am. Diet. Assoc..

[18] Chumlea W.C., Roche A.F., Steinbaugh M.L. (1985). Estimating stature from knee height for persons 60 to 90 years of age. J. Am. Geriatr. Soc..

[19] Prothro J.W., Rosenbloom C.A. (1993). Physical measurements in an elderly black population: Knee height as the dominant indicator of stature. J. Gerontol..

[20] Ahmad N., Mohammad F., Farooque I. (2014). ESTIMATION OF HEIGHT FROM THE LONG BONES OF UPPERLIMB AND HAND DIMENSIONS IN SOUTH INDIAN POPULATION. J. Evid. Based Med. Healthc..

[21] Bassey E.J. (1986). Demi-span as a measure of skeletal size. Ann. Hum. Biol..

[22] Hirani V., Tabassum F., Aresu M., Mindell J. (2010). Development of new demi-span equations from a nationally representative sample of adults to estimate maximal adult height. J. Nutr..

[23] Guerra R.S., Fonseca I., Pichel F., Restivo M.T., Amaral T.F. (2014). Hand length as an alternative measurement of height. Eur. J. Clin. Nutr..

[24] Reeves S.L., Varakamin C., Henry C.J. (1996). The relationship between arm-span measurement and height with special reference to gender and ethnicity. Eur. J. Clin. Nutr..

[25] de Lucia E., Lemma F., Tesfaye F., Demisse T., Ismail S. (2002). The use of armspan measurement to assess the nutritional status of adults in four Ethiopian ethnic groups. Eur. J. Clin. Nutr..

[26] Elia M. (2003). The ‘MUST’ Report: Nutritional Screening of Adults: A Multidisciplinary Responsibility: Development and Use of the ‘Malnutrition Universal Screening Tool’ (‘MUST’) for Adults.

[27] Chumlea W.C., Guo S.S., Wholihan K., Cockram D., Kuczmarski R.J., Johnson C.L. (1998). Stature prediction equations for elderly non-Hispanic white, non-Hispanic black, and Mexican-American persons developed from NHANES III data. J. Am. Diet. Assoc..

[28] Launer L.J., Harris T. (1996). Weight, height and body mass index distributions in geographically and ethnically diverse samples of older persons. Ad Hoc Committee on the Statistics of Anthropometry and Aging. Age Ageing.

[29] WHO Working Group on the Purpose, Use and Interpretation of Anthropometric Indicators of Nutritional Status (1995). Physical status: The use and interpretation of anthropometry. Report of a WHO Expert Committee. World Health Organ. Tech. Rep. Ser..

[30] Cline M.G., Meredith K.E., Boyer J.T., Burrows B. (1989). Decline of height with age in adults in a general population sample: Estimating maximum height and distinguishing birth cohort effects from actual loss of stature with aging. Hum. Biol..

[31] Madden A.M., Mashanova A., Amirabdollahian F., Ghuman S., Makda M., Collinson A., Dean F., Hirsz M., Lennie S., Maynard M.J. (2020). Improved prediction equations for estimating height in adults from ethnically diverse backgrounds. Clin. Nutr..

[32] Williamson H., Walsh C., Nel M., van den Berg-van Antwerpen V. (2022). Agreement between measured height, and height predicted from published equations, in adult South African patients. South Afr. J. Clin. Nutr..

[33] Madden A.M., Tsikoura T., Stott D.J. (2012). The estimation of body height from ulna length in healthy adults from different ethnic groups. J. Hum. Nutr. Diet..

[34] Venkataraman R., Ranganathan L., Nirmal V., Kameshwaran J., Sheela C.V., Renuka M.V., Ramakrishnan N. (2015). Height measurement in the critically ill patient: A tall order in the critical care unit. Indian. J. Crit. Care Med..

[35] Pini R., Tonon E., Cavallini M.C., Bencini F., Di Bari M., Masotti G., Marchionni N. (2001). Accuracy of equations for predicting stature from knee height, and assessment of statural loss in an older Italian population. J. Gerontol. A Biol. Sci. Med. Sci..

[36] Perkins J.M., Subramanian S.V., Davey Smith G., Özaltin E. (2016). Adult height, nutrition, and population health. Nutr. Rev..

